# Polymorphisms in *α*- and *β*-Adrenergic Receptor Genes, Hypertension, and Obstructive Sleep Apnea: The Skaraborg Sleep Study

**DOI:** 10.4061/2010/458410

**Published:** 2010-03-30

**Authors:** Kristina Bengtsson Boström, Jan Hedner, Ludger Grote, Olle Melander, Fredrik von Wowern, Lennart Råstam, Leif Groop, Ulf Lindblad

**Affiliations:** ^1^R&D Centre Skaraborg Primary Care, Storgatan 18, 541 30 Skövde, Sweden; ^2^Department of Clinical Sciences, Diabetes and Endocrinology, Malmö University Hospital, Lund University, 20502 Malmö, Sweden; ^3^Department of Clinical Sciences, Community Medicine, Malmö University Hospital, Lund University, 20502 Malmö, Sweden; ^4^Sleep Laboratory, The Sahlgrenska Academy, University of Gothenburg, 41345 Gothenburg, Sweden; ^5^Department of Public Health and Community Medicine/Primary Health Care, The Sahlgrenska Academy, University of Gothenburg, P.O. Box 400, 405 30 Göteborg, Sweden; ^6^Skaraborg Institute, Stationsgatan 12, 54130 Skövde, Sweden

## Abstract

The sympathetic nervous system and the adrenergic receptors play an important role in regulation of blood pressure. This study explored the associations between functional polymorphisms of the *α*_2B_-, *β*_1_-, and *β*_2_-adrenergic receptor genes and obstructive sleep apnea (OSA) in hypertensive patients and hypertension in patients with OSA in a populationbased sample of 157 hypertensive patients and 181 healthy control subjects. Only the Arg389Gly polymorphism of the *β*_1_-adrenergic receptor gene was associated with increased risk for mild OSA in hypertensive patients (Arg/Arg versus Gly/Arg/Gly/Gly, 2.1, 95% CI, 1.02–4.7). Hypertensive men carrying the Arg389Arg genotype had higher crude and age-adjusted AHI than carriers of the Arg389Gly/Gly389Gly genotypes. When adjusted also for BMI this difference became borderline significant. This difference was not observed in women. The risk of hypertension in mild OSA was associated with increasing number of Arg-alleles (Arg/Arg OR 5.4, 95% CI 1.4–21.2).

## 1. Introduction

Obstructive sleep apnea (OSA) is a disorder characterized by upper airway collapse and is common in middle-aged adults [1]. OSA often leads to hypoxia and activation of the sympathetic nervous system [2, 3] which might lead to adverse metabolic reactions and high blood pressure [4, 5]. OSA has in humans been associated with hypertension [6, 7] and cardiovascular complications [8, 9]. In an earlier study of a population-based sample of hypertensive patients and normotensive controls from primary care in Sweden, OSA was found to be more prevalent in hypertensive patients, specifically in male patients compared to controls, 83% versus 64% (58% versus 49% in females) when OSA was defined as apnea/hypopnea index (AHI) ≥ 10 events per hour and 47% versus 25% (26% versus 24% in females) when OSA was defined as AHI ≥ 30 events per hour [7].

The sympathetic nervous system plays an important role in regulation of blood pressure and the adrenergic receptors are important components of this system. The aim of this study was to explore the associations between functional polymorphisms of the *α*_2B_-, *β*_1_-, and *β*_2_-adrenergic receptor genes and occurrence of OSA in hypertensive patients and occurrence of hypertension in patients with OSA in a population-based sample. 

The *α*_2A_-adrenergic receptor and the *α*_2B_-adrenergic receptor are expressed in the central nervous system. Blood pressure is regulated by the opposing action of these receptors upon agonist binding. The *α*_2A_-adrenergic receptor has an inhibiting effect on the sympathetic outflow while the *α*_2B_-adrenergic receptor has an excitatory effect [10]. The *α*_2B_-adrenergic receptor gene is located on chromosome 2 in a region where several genome scans, including one from the population in the current study [11], have found linkage to blood pressure variation and hypertension [12–14]. In the third intracellular loop of the receptor, in an area of importance in downregulation, there is a polymorphism consisting of either an insertion (I) or a deletion (D) of three amino acids at positions 301–303. Earlier studies have shown that the deletion variant confers an increased activity in the sympathetic nervous system [15] and that it is associated with hypertension in the current and other populations [16, 17].

The *β*_1_-adrenergic receptor is expressed in cardiac myocytes [18] and upon agonist binding it confers excitatory reactions in the myocyte leading to higher cardiac output through increased cardiac inotropy and chronotropy.

The *β*_1_-adrenergic receptor gene on chromosome 10 harbors two polymorphisms with functional properties. The Ser49Gly, located in the extra cellular portion of the receptor, has been associated with variations in resting heart rate in a dose dependent way [19]. The other polymorphism, Arg389Gly, is located in the intracellular cytoplasmic tail of the receptor near the seventh transmembrane region, where the stimulatory G-protein probably binds. In vitro studies have shown that the Arg389 variant mediates a higher isoproterenol stimulated adenylate cyclase activity than the Gly389 variant [20]. Homozygous carriers of Arg389 have been shown to have a higher risk for hypertension and higher diastolic blood pressure levels and heart frequency than carriers of the other genotypes in the current population [21] and a minor influence on both systolic and diastolic blood pressure in a larger population [22]. The homozygous Arg389 carriers have also been shown to have a greater blood pressure lowering response to atenolol [23] and metoprolol [24] compared with the homozygous Gly389 carriers strengthening its functional importance in cardiovascular regulation. Whether the polymorphism is associated with higher risk for cardiovascular complications is not clear [25, 26]. Recently, it has been shown that the Arg389Gly polymorphism can modify the beneficial effect of continuous airway pressure [27].

The *β*_2_-adrenergic receptor is expressed in most arteries in the body and in the heart and also in the respiratory tract. Upon agonist binding it elicits dilation of the arteries and bronchi. The *β*_2_-adrenergic receptor gene is located on chromosome 5 in an area where already in 1996 genetic variation was found to be associated with hypertension [28]. Linkage to blood pressure levels has been found in genome wide scans [29] and subsequent fine mapping has revealed association between hypertension and the *β*_2_-adrenergic receptor gene variants [30] in the cytoplasmic tail of the receptor. Two polymorphisms, the Arg16Gly and Gln27Glu, have been found in vitro to confer different degree of downregulation of the receptor [31]. Studies on humans have given conflicting results possibly because the two polymorphisms have different properties and are in linkage disequilibrium with each other [32]. In the Ectim and PEGASE studies there was no association between the polymorphisms of the receptor and myocardial infarction [33]. In studies of Scandinavian subjects, the Arg16 and Gln27 variants were found to be associated with an increased risk of hypertension [34] and with hypertension associated with type 2 diabetes [35].

## 2. Subjects and Methods

### 2.1. Study Population

The Skaraborg Hypertension and Diabetes Project cohort have previously been described in detail [36]. This sample comprises the majority of patients with hypertension and/or type 2 diabetes (*n* = 1149) in the local community of Skara investigated in 1992-1993 and an age-stratified, random sample from 40 years of age from Skara population (*n* = 1109) that was investigated in 1993-1994 [37]. In 2000, a random sample of hypertensive and normotensive men and women from 40 to 64 years at the baseline investigations was drawn from the previous cohorts. The diagnosis of hypertension was based upon at least three consecutive diastolic blood pressure measurements ≥90 mmHg diastolic blood pressure (DBP) according to the current National Guidelines [38, 39] or ongoing antihypertensive treatment at baseline. Most hypertensive subjects in the present study (at least 85%) were already diagnosed according to the criteria used before 1989 (≥160/95 mmHg, WHO, 1989 [40]) and had ongoing antihypertensive treatment. Normotension was defined as a systolic blood pressure (SPB) < 160 mmHg and a DBP < 90 mmHg and no antihypertensive medication. Type 2 diabetes was diagnosed according to the current WHO recommendations [41] in 21 hypertensive patients. A full-night polysomnography (PSG) at home was performed in 344 subjects, 161 patients with hypertension, and 181 individuals without hypertension. The local ethics committee approved the study and written informed consent was obtained from all the participants.

### 2.2. Study Procedure

 At baseline 1992–1994 all subjects were surveyed for cardiovascular disease (CVD) risk factors using a previously published protocol [42]. Sleep studies were conducted during 2000–2002. A specially trained study nurse performed the basic investigations. The blood pressure was measured with Tricuff [43] in the supine position with a sphygmomanometer after 5 minutes of rest. A standardised interview on medical history and medications was performed and a questionnaire on sleep quality, daytime sleepiness, and symptoms of depression was administered [44]. Height was measured to the nearest centimetre and weight to the nearest 0.1 kg and body mass index (BMI) was calculated. A full night PSG recording was performed in the subjects' home using a portable Embla-A10 polysomngraphy system (Flaga, Reykjavik, Iceland). Each recording included three-channel electroencephalography (EEG, C_4_/A_1_, C_3_/A_2_), two-channel electrooculography, and one-channel submental electromyography (EMG). Limb movements were monitored by tibial surface electrodes. Ventilatory monitoring included oxygen saturation (SaO_2_) by an oximeter probe, respiratory movements by chest and abdominal belts, nasal and oral airflow by thermistor and nasal pressure by pressure sensors. Two cotrained staff members manually scored the recordings according to a validated scoring manual [45]. A successful PSG required a minimum of 4 hours sleeping time including at least one REM-sleep period. The number of apnea/hypopnea events per hour of sleep (AHI) was used to dichotomise subjects into two groups, one with a cutoff level of 10 events per hour (mild OSA, OSA10, AHI ≥ 10 events per hour) and one with a cutoff level of 30 events per hour (severe OSA, OSA30, AHI ≥ 30 events per hour). Separate statistical analyses were undertaken to compare the two groups with the control subjects (those with AHI <10 and <30 events per hour, resp.).

### 2.3. Genotyping

High molecular weight DNA was extracted from blood leukocytes according to standard techniques [46] and used as template in all polymerase chain reactions (PCRs). The genotyping procedures have been described elsewhere [16, 35, 47]. The PCR conditions and digestion conditions in the restriction fragment length polymorphism (RFLP) reactions are shown in Table 1.

### 2.4. Statistical Methods

Hypertensive and normotensive subjects were analysed separately. Continuous variables are presented as means ±  standard deviation (SD) and discrete variables as frequencies (%). Differences between means were tested by ANCOVA. Differences in proportions were tested by *χ* ^2^-test and by logistic regression. Confounding from age and sex was accounted for by multivariate analyses or by stratification. Multiple logistic regressions were performed with mild OSA (OSA10) and severe OSA (OSA30) as the dependent variable and with age, sex, BMI and the *α*_2B_-adrenergic receptor, *β*_1_- and *β*_2_-adrenergic receptor polymorphisms as independent variables. Associations were expressed as odds ratio (OR) with 95 per cent confidence interval (95% CI). For the *α*_2B_-adrenergic receptor gene the II genotype was used as reference in the comparison with the ID and DD genotypes. For the *β*_1_-adrenergic receptor gene the combination of Ser49Gly and Gly49Gly was used as a reference and compared with the Ser49Ser genotype. The combination of Gly389Gly and Arg389Gly genotypes was used as reference and compared with the Arg389Arg genotype. Finally, for the *β*_2_-adrenergic receptor gene the Gly16Gly and Glu27Glu genotypes were used as references compared with the Arg16Gly and Arg16Arg and the Gln27Glu and Gln27Gln genotypes, respectively. Analyses were carried out using SPSS Base system for Macintosh 11.0 (Chicago, SPSS Inc.). All statistical tests were two sided. *P*-values less than.05 were considered statistically significant.

## 3. Results

The clinical characteristics of the subjects are shown in Table 2. As expected hypertensive patients had higher systolic and diastolic blood pressure but were also older and had higher BMI than normotensive subjects. AHI was higher in hypertensive men but did not differ between hypertensive and normotensive women. The observed genotype frequencies were all in Hardy Weinberg equilibrium.

### 3.1. The *α*_2B_-Adrenergic Receptor

The genotype frequencies of the *α*_2B_-adrenergic receptor gene were 15% for DD genotype, 51% for ID genotype, and 34% for the II genotype. There was no statistical difference in genotype or allele frequencies of the 301–303 I/D polymorphism in hypertensive patients with or without OSA (Table 3) or in normotensive controls (data not shown).

### 3.2. The *β*_1_-Adrenergic Receptor

The Arg389Arg genotype of the *β*_1_-adrenergic receptor gene was more common in male hypertensive patients with mild OSA than in those without mild OSA (Table 3). There was a similar trend in hypertensive females and in hypertensive males with severe OSA. Among the 62 analyzed men there was no one with OSA carrying the Gly389Gly genotype (Table 3). In a recessive model comparing Arg389Arg to Arg389Gly and Gly389Gly there was a significant association with mild OSA in hypertensive subjects, OR 2.3 (95% CI 1.10–4.87), *P* = .027, crude and when adjusted for age and sex OR 2.3 (95% CI 1.09–4.96), *P* = .029 and still significant when adjusted also for BMI OR 2.1 (95% CI 1.02–4.71), *P* = .045. In sex specific analysis the patterns were very similar in men and women; however, neither crude nor adjusted models turned out significant. In the full model we found OR 3.4 (95% CI 0.85–13.40), *P* = .085 in men, and 2.26 (95% CI 0.85–5.99), *P* = .102 in women, respectively. Hypertensive men carrying the Arg389Arg genotype had higher crude and age-adjusted AHI than carriers of the Arg389Gly/Gly389Gly genotypes. When adjusted also for BMI this difference became borderline significant. This difference was not observed in women (Table 4). There was also a dose dependent increase in AHI according to genotype being the lowest in Gly389Gly carriers increasing in the Arg389Gly and the highest in Arg389Arg carriers, significant in all men (15.0 ± 13.7, 24.3 ± 18.5 and 32.3 ± 26.2 events per hour, *P* = .017). In the subgroups of men with and without hypertension AHI showed a similar increasing trend, 4.0 (one subject), 27.2 ± 20.1 and 39.5 ± 27.7 events per hour, *P* = .065 and 16.1 ± 13.9, 21.8 ± 16.9, and 25.9 ± 23.4 events per hour, *P* = .33, respectively. In women no such trend was observed (28.8 ± 27.5, 22.9 ± 25.3, and 23.7 ± 23.1 events per hour *P* = .72). 

In analysis including both patients with hypertension and normotensive controls subjects the frequency of hypertension versus normotension differed significantly between the different genotype carriers in subjects with mild OSA (Table 5). When using the number of Arg389 alleles as an approximately linear variable the age and BMI adjusted OR for hypertension in patients with mild OSA were for one Arg389 allele 4.8 (95% CI 1.2–19.3), *P* = .028 and for two Arg389 alleles 5.4 (95% CI 1.4–21.2), *P* = .015. A test for trend adjusted for age, sex, and BMI also showed an increased OR for hypertension with increasing number of Arg389 alleles, OR 1.6, 95% CI 1.003–2.5, *P* = .048.

### 3.3. The *β*_2_-Adrenergic Receptor

Neither of the investigated polymorphisms (Arg16Gly or Gln27Glu) was associated with increased risk of OSA in hypertensive subjects or with hypertension in patients with OSA (data not shown).

## 4. Discussion

We describe an association between the Arg389Arg genotype of the *β*_1_-adrenergic receptor gene and OSA in hypertensive patients. Furthermore, occurrence of hypertension increased in subjects with mild OSA with increasing number of Arg389 alleles. The Arg389Arg genotype carriers had a borderline significantly higher AHI than the Arg389Gly/Gly389Gly carriers when adjusted for age and BMI. While the current study was designed to investigate the risk of OSA in patients with hypertension, the results showing that the risk of hypertension in subjects with mild OSA was associated with the Arg389 allele are in accordance with what have been previously published on association between hypertension and the Arg389Gly polymorphism [21]. 

The *β*_1_-adrenergic receptor is important in the adrenergic activation of the heart by increasing both the inotropic and chronotropic responses upon agonist binding. The Arg389 variant of the *β*_1_-adrenergic receptor has been found to increase both the basal and the isoprotenerol-stimulated adenylate cyclase activity compared with the Gly389 variant. Experimental hypoxia in rats is a powerful activator of the sympathetic nervous system leading to increased blood pressure [48]. This is consistent with our results that a variant of the *β*_1_-adrenergic receptor that confers an increased cyclic AMP activity and has been shown to be associated with hypertension also could be associated with increased risk of OSA in hypertensive patients. The gender difference found in this study could be due to the lower impact of the genetic variants of the sympathetic nervous system in development of OSA in females compared with males; instead anatomic factors in the throat might play a greater role in females [49]. The analysis of AHI in different *β*_1_-adrenergic receptor genotype carriers showed a significant difference only in males. In fact we found no difference in AHI between female hypertensive patients and controls. This suggests that hypertension plays a less important role in OSA in women than in men. One limitation of this study is the small number of subjects especially after dichotomization into hypertension and normotension. In this particular polymorphism where the Gly389Gly genotype has a low frequency the data should be interpreted with care. 

 The polymorphisms in the other adrenergic receptor genes (the *α*_2B_-adrenergic receptor and the *β*_2_-adrenergic receptor) were not associated with OSA in this study population.

The *α*_2B_-adrenergic receptor gene I/D 301–303 polymorphism studied here is proposed to influence the agonist-induced desensitization of the receptor. We found no association between this polymorphism and OSA in hypertensive or normotensive individuals. One explanation might be that the *α*_2B_-adrenergic receptor acts on a level of sympathetic action that is only indirectly involved in the adrenergic response of the heart to hypoxia induced during OSA.

The lack of association between polymorphisms of the *β*_2_-adrenergic receptor and OSA is in accordance with the results from a cohort with high prevalence of hypertension from Germany [50] although the risk of myocardial infarctions was associated with the polymorphisms. Furthermore, these polymorphisms have been shown to be associated with hypertension only in the presence of type 2 diabetes in the current population [35]. Compared to the *β*_1_-adrenergic receptor the *β*_2_-adrenergic receptor has a similar but less pronounced effect in the heart. This difference in action could explain why the *β*_2_-polymorphisms investigated here do not seem to be of importance in development of OSA in hypertensive patients. 

In conclusion, the Arg389Gly polymorphism of the *β*_1_-adrenergic receptor was associated with increased risk of OSA in hypertensive men and with hypertension in subjects with mild OSA. Also, in men, there was a dose dependent increase in AHI being the lowest in Gly389Gly carriers and the highest in Arg389Arg carriers. These findings highlight the role of the sympathetic nervous system in OSA and hypertension.

## Figures and Tables

**Table 1:** PCR and digestion conditions for genotyping of the the *α*_2*B*_-, *β*_1_-, and the *β*_2_-adrenergic receptor gene polymorphisms.

		PCR conditions					RFLP		Gel^‡‡^
Gene/polymorphism	Forward (sense) primer sequence								
N successfully genotyped	Reverse (antisense) primer sequence	Size (bp)	Annealing* T* (°C)	Extension time (s)	Mg^2^+ mmol/L	Cycles	Enzyme	Time (h)	%
338 A2BAR									
I/D 301–303	5′-AGGGTGTTTGTGGGGCATCT	112/103	72	30	1.5	33	—		3
	5′-CAAGCTGAGGCCGGAGACACT								
337* B1AR									
Ser49Gly	5′-CCGGGCTTCTGGGGTGTTCC	562	64	30	1.5	35	*EcoO109I*	3	2
	5′-GGCGAGGTGATGGCGAGGTAGC								
Arg389Gly	5′-CGCTCTGCTGGCTGCCCTTCTTCC	530	64	30	1.5	33	*BcgI*	3	2
	5′-TGGGCTTCGAGTTCACCTGCTATC								
338 B2AR									
Arg16Gly	5′-CGCCTTCTTGCTGGCACGCAAT**	203	60	30	1.5	30	*BsrDl*	2	4.5
	5′-CCAGTGAAGTGATGAAGTAGTT								
Gln27Glu	5′-CCGGACCACGACGTCACCCAG***	169	60	30	1.5	30	*BstNl*	2	4.5
	5′-CCAGTGAAGTGATGAAGTAGTT								

A2BAR: *α*_2B_-adrenergic receptor gene; B1AR: *β*_1_-adrenergic receptor; B2AR: *β*_2_-adrenergic receptor; Ag: agarose; bp: base pair.

*The genotyping failed in one individual.

**The underlined nucleotide is a mismatch to create a *BsrDl* recognition site in case of the Gly16-allele.

***The underlined nucleotide is a mismatch to create a *BstNl* recognition site in case of the Glu27-allele.

^‡‡^The PCR products were separated on a multipurpose agarose gel (Ag) with ethidium bromide and visualised with UV-light.

**Table 2:** Clinical characteristics in normotensive and hypertensive men and women.

	Normotensive subjects		Patients with hypertension		
Variables	Mean	(SD)	Mean	(SD)	*P*
*Men (n)*	*96*		*74*		
Age (years)	60	(6.3)	62	(6.2)	.008
Systolic blood pressure (mmHg)	131	(13.2)	148	(15.7)	<.001
Diastolic blood pressure (mmHg)	77	(7.5)	84	(6.7)	<.001
Heart rate (beats per minute)	68	(8.3)	68	(8.2)	.653
AHI (events per hour)	23	(20.4)	34	(25.6)	.005
Body mass index (kg/m^2^)	26.8	(3.0)	29.5	(4.6)	<.001
*Women (n)*	*85*		*83*		
Age (years)	60	(7.1)	64	(5.7)	<.001
Systolic blood pressure (mmHg)	132	(14.8)	150	(16.8)	<.001
Diastolic blood pressure (mmHg)	75	(7.5)	83	(8.0)	<.001
Heart rate (beats per minute)	70	(8.7)	69	(9.0)	.944
AHI (events per hour)	22	(26.0)	25	(22.2)	.655
Body mass index (kg/m^2^)	28.1	(4.8)	30.3	(5.7)	.006

Data are mean (SD). Differences in means were analysed by general linear models with adjustment for age. AHI: apnea/hypopnea index.

**Table 3:** Genotype frequencies of adrenergic receptor genes: *α*_2*B*_-adrenergic I/D, the *β*_1_ Ser49Gly and Gly389Arg, and the *β*_2_ Gly16Arg and Gln27Glu polymorphisms in hypertensive patients with and without mild (panel A) and severe (panel B) obstructive sleep apnoea (OSA).

A	Patients with mild OSA			Patients without mild OSA			*P*
*α*_2B_ *-adrenergic receptor gene I/D polymorphism *(*n* = 157)							
Genotype	II	ID	DD	II	ID	DD	

*Men* OSA10 (*n* = 74)	18 (28.6)	32 (50.8)	13 (20.6)	5 (41.7)	6 (50.0)	1 (8.3)	.50
*Women *OSA10 (*n* = 83)	20 (35.7)	27 (48.2)	9 (16.1)	9 (34.6)	11 (42.3)	6 (23.1)	.73

*β*_1_ - *adrenergic receptor gene Ser49Gly and Gly389Arg polymorphisms *(*n* = 156)							
Genotype (Ser49Gly)	S/S	S/G	G/G	S/S	S/G	G/G	*P*

*Men* OSA10 (*n* = 74)	37 (59.7)	24 (38.7)	1 (1.6)	9 (75.0)	3 (25.0)	0 (0.0)	.58
*Women *OSA10 (*n* = 82)	39 (69.6)	12 (21.4)	5 (8.9)	20 (76.9)	6 (23.1)	0 (0.0)	.29

Genotype (Gly389Arg)	G/G	G/A	A/A	G/G	G/A	A/A	*P*

*Men* OSA10 (*n* = 74)	0 (0.0)	23 (37.1)	39 (62.9)	1 (8.3)	6 (50.0)	5 (41.7)	.042
*Women *OSA10 (*n* = 82)	3 (5.4)	18 (32.1)	35 (62.5)	3 (11.5)	12 (46.2)	11 (42.3)	.20

*β*_2_ * adrenergic receptor Gly16Arg and Gln27Glu *(*n* = 157)							
Genotype (Gly16Arg)	A/A	A/G	G/G	A/A	A/G	G/G	*P*

*Men* OSA10 (*n* = 74)	8 (12.9)	35 (56.5)	19 (30.6)	1 (8.3)	5 (41.7)	6 (50.0)	.43
*Women *OSA10 (*n* = 83)	8 (14.0)	22 (38.6)	27 (47.4)	4 (15.4)	13 (50.0)	9 (34.6)	.54

Genotype (Gln27Glu)	Gn/Gn	Gn/Gu	Gu/Gu	Gn/Gn	Gn/Gu	Gu/Gu	*P*

*Men* OSA10 (*n* = 74)	24 (38.7)	28 (45.2)	10 (16.1)	1 (8.3)	8 (66.7)	3 (25.9)	.13
*Women *OSA10 (*n* = 83)	17 (29.8)	27 (47.4)	13 (22.8)	10 (38.5)	10 (38.5)	6 (23.1)	.70

B	Patients with severe OSA			Patients without severe OSA			

*α*_2B_ *-adrenergic receptor gene I/D polymorphism *(*n* = 157)							
Genotype		II	ID	DD	II	ID	*P*

*Men* OSA30 (*n* = 74)	12 (34.3)	15 (42.9)	8 (22.9)	11 (27.5)	23 (57.5)	6 (15.0)	.43
*Women *OSA30 (*n* = 83)	8 (36.4)	12 (54.6)	2 (9.1)	21 (35.0)	26 (43.3)	13 (21.7)	.40

**β*_1_ adrenergic receptor gene Ser49Gly and Gly389Arg polymorphisms *(*n* = 156)							
Genotype (Ser49Gly)	S/S	S/G	G/G	S/S	S/G	G/G	*P*

*Men* OSA30 (*n* = 74)	24 (68.6)	11 (31.4)	0 (0.0)	22 (56.4)	16 (41.0)	1 (2.6)	.41
*Women* OSA30 (*n* = 82)	15 (68.2)	5 (22.7)	2 (9.1)	44 (73.3)	13 (21.7)	3 (5.0)	.78

Genotype (Gly389Arg)	G/G	G/A	A/A	G/G	G/A	A/A	*P*

*Men *OSA30 (*n* = 74)	0 (0.0)	10 (28.6)	25 (71.4)	1 (2.6)	19 (48.7)	19 (48.7)	.11
*Women* OSA30 (*n* = 82)	2 (9.1)	7 (31.8)	13 (59.1)	4 (6.7)	23 (38.3)	33 (55.0)	.83

**β*_2 _adrenergic receptor Gly16Arg and Gln27Glu *(*n* = 157)							
Genotype (Gly16Arg)	A/A	A/G	G/G	A/A	A/G	G/G	*P*

*Men* OSA30 (*n* = 74)	2 (5.7)	22 (62.9)	11 (31.4)	7 (18.0)	18 (46.2)	14 (35.9)	.19
*Women* OSA30 (*n* = 83)	4 (18.2)	8 (36.4)	10 (45.5)	8 (13.1)	27 (44.3)	26 (42.6)	.76

Genotype (Gln27Glu)	Gn/Gn	Gn/Gu	Gu/Gu	Gn/Gn	Gn/Gu	Gu/Gu	*P*

*Men* OSA30 (*n* = 74)	11 (31.4)	19 (54.3)	5 (14.3)	14 (35.9)	17 (43.6)	8 (20.5)	.62
*Women* OSA30 (*n* = 83)	6 (27.3)	10 (45.5)	6 (27.3)	21 (34.4)	27 (44.3)	13 (21.3)	.77

OSA10: subjects dichotomised by AHI < or ≥10 events/ hour; OSA30: subjects dichotomised by AHI < or ≥30 events/hour. *P*-values are estimated with chi-2 test.

A: (Arg) arginine; D: deletion; I: insertion; G: (Gly) Glycine; Gn:(Gln) Glutamine; Gu: (Glu) glutamic acid; S:(Ser) serine; HT: hypertensive; OSA: obstructive sleep apnoea.

**Table 4:** Differences in AHI between male and female carriers of the Arg389Gly genotypes of the Beta-1 adrenergic gene.

	Gly389Gly/Arg389Gly		Arg389Arg		
AHI (events per hour)	Mean	(SE)	Mean	(SE)	*P* *(adjusted)*
*Males*	*n* = 96		*n* = 74		

Normotensive subjects					
(crude)	20.5	(3.0)	25.9	(2.9)	.20
(adjusted for age)	20.1	(3.1)	26.3	(2.9)	.15
(adjusted for age, BMI)	20.5	(3.0)	25.9	(2.8)	.20
Hypertensive patients					
(crude)	26.5	(4.6)	39.5	(3.8)	.03
(adjusted for age)	26.3	(4.6)	39.6	(3.8)	.03
(adjusted for age, BMI)	27.4	(4.5)	38.6	(3.7)	.06

*Females*	*n* = 85		*n* = 82		

Normotensive subjects					
(crude)	23.5	(4.1)	21.3	(3.9)	.70
(adjusted for age)	23.4	(3.9)	21.3	(3.8)	.70
(adjusted for age, BMI)	22.8	(3.7)	21.9	(3.5)	.85
Hypertensive patients					
(crude)	24.4	(3.7)	26.0	(3.3)	.76
(adjusted for age)	23.9	(3.6)	26.3	(3.1)	.61
(adjusted for age, BMI)	24.3	(3.6)	26.1	(3.1)	.71

AHI: apnoea-hypopnoea index (events per hour). Data are mean (SE) and stepwise adjusted for age and age and BMI. One male hypertensive had missing BMI.

**Table 5:** Frequency of hypertension in patients with mild obstructive sleep apnea according to Beta-1 adrenergic receptor Arg389Gly polymorphism.

Beta-1 adrenergic Genotype	Hypertension	Normotension	*P*
*All (* *n* = 229)	*n* = 118	*n* = 111	

Gly389Gly%	2.5	9.9	
Gly389Arg%	34.8	37.8	
Arg389Arg%	62.7	52.3	.043

*Males (* *n* = 124)	*n* = 62	*n* = 62	

Gly389Gly%	0	9.7	
Gly389Arg%	37.1	37.1	
Arg389Arg%	62.9	53.2	.039

*P*-values are estimated with chi-2 test.

## References

[1] Pepperell JCT, Davies RJO, Stradling JR (2002). Systemic hypertension and obstructive sleep apnoea. *Sleep Medicine Reviews*.

[2] Carlson JT, Hedner J, Elam M, Ejnell H, Sellgren J, Wallin BG (1993). Augmented resting sympathetic activity in awake patients with obstructive sleep apnea. *Chest*.

[3] Loredo JS, Ziegler MG, Ancoli-Israel S, Clausen JL, Dimsdale JE (1999). Relationship of arousals from sleep to sympathetic nervous system activity and BP in obstructive sleep apnea. *Chest*.

[4] Fletcher EC, Bao G, Li R (1999). Renin activity and blood pressure in response to chronic episodic hypoxia. *Hypertension*.

[5] Newman AB, Nieto FJ, Guidry U (2001). Relation of sleep-disordered breathing to cardiovascular disease risk factors: the Sleep Heart Health Study. *American Journal of Epidemiology*.

[6] Peppard PE, Young T, Palta M, Skatrud J (2000). Prospective study of the association between sleep-disordered breathing and hypertension. *New England Journal of Medicine*.

[7] Hedner J, Bengtsson-Boström K, Peker Y, Grote L, Råstam L, Lindblad U (2006). Hypertension prevalence in obstructive sleep apnoea and sex: a population-based case—control study. *European Respiratory Journal*.

[8] Nieto FJ, Young TB, Lind BK (2000). Association of sleep-disordered breathing sleep apnea, and hypertension in a large community-based study. *Journal of the American Medical Association*.

[9] Shahar E, Whitney CW, Redline S (2001). Sleep-disordered breathing and cardiovascular disease: cross-sectional results of the Sleep Heart Health Study. *American Journal of Respiratory and Critical Care Medicine*.

[10] Gavras I, Manolis AJ, Gavras H (2001). The *α*2-adrenergic receptors in hypertension and heart failure: experimental and clinical studies. *Journal of Hypertension*.

[11] von Wowern F, Bengtsson K, Lindgren CM (2003). A genome wide scan for early onset primary hypertension in Scandinavians. *Human Molecular Genetics*.

[12] Rice T, Rankinen T, Province MA (2000). Genome-wide linkage analysis of systolic and diastolic blood pressure: the Québec Family Study. *Circulation*.

[13] Perola M, Kainulainen K, Pajukanta P (2000). Genome-wide scan of predisposing loci for increased diastolic blood pressure in Finnish siblings. *Journal of Hypertension*.

[14] Kristjansson K, Manolescu A, Kristinsson A (2002). Linkage of essential hypertension to chromosome 18q. *Hypertension*.

[15] Suzuki N, Matsunaga T, Nagasumi K (2003). *α*2b-Adrenergic receptor deletion polymorphism associates with autonomic nervous system activity in young healthy Japanese. *Journal of Clinical Endocrinology and Metabolism*.

[16] Von Wowern F, Bengtsson K, Lindblad U (2004). Functional variant in the *α*2B adrenoceptor gene, a positional candidate on chromosome 2, associates with hypertension. *Hypertension*.

[17] Vasudevan R, Ismail P, Stanslas J, Shamsudin N, Ali AB (2008). Association of insertion/deletion polymorphism of alpha-adrenoceptor gene in essential hypertension with or without type 2 diabetes mellitus in Malaysian subjects. *International Journal of Biological Sciences*.

[18] Strader CD, Fong TM, Tota MR, Underwood D, Dixon RAF (1994). Structure and function of G protein-coupled receptors. *Annual Review of Biochemistry*.

[19] Ranade K, Jorgenson E, Sheu WH-H (2002). A polymorphism in the *β*1 adrenergic receptor is associated with resting heart rate. *American Journal of Human Genetics*.

[20] Mason DA, Moore JD, Green SA, Liggett SB (1999). A gain-of-function polymorphism in a G-protein coupling domain of the human *β*
_1_-adrenergic receptor. *Journal of Biological Chemistry*.

[21] Bengtsson K, Melander O, Orho-Melander M (2001). Polymorphism in the *β*1-adrenergic receptor gene and hypertension. *Circulation*.

[22] Gjesing AP, Andersen G, Albrechtsen A (2007). Studies of associations between the Arg389Gly polymorphism of the *β*
_1_-adrenergic receptor gene (ADRB1) and hypertension and obesity in 7677 Danish white subjects. *Diabetic Medicine*.

[23] Sofowora GG, Dishy V, Muszkat M (2003). A common *β*1-adrencrgic receptor polymorphism (Arg389Gly) affects blood pressure response to *β*-blockade. *Clinical Pharmacology and Therapeutics*.

[24] Johnson JA, Zineh I, Puckett BJ, McGorray SP, Yarandi HN, Pauly DF (2003). *β*
_1_-adrenergic receptor polymorphisms and antihypertensive response to metoprolol. *Clinical Pharmacology and Therapeutics*.

[25] White HL, Maqbool A, McMahon AD (2002). An evaluation of the beta-1 adrenergic receptor Arg389Gly polymorphism in individuals at risk of coronary events. A WOSCOPS substudy. *European Heart Journal*.

[26] Iwai C, Akita H, Kanazawa K (2003). Arg389Gly polymorphism of the human *β*
_1_-adrenergic receptor in patients with nonfatal acute myocardial infarction. *American Heart Journal*.

[27] Börgel J, Schulz T, Bartels NK (2006). Modifying effects of the R389G *β*
_1_-adrenoceptor polymorphism on resting heart rate and blood pressure in patients with obstructive sleep apnoea. *Clinical Science*.

[28] Svetkey LP, Timmons PZ, Emovon O, Anderson NB, Preis L, Chen Y-T (1996). Association of hypertension with B_2_-and A_2c10_-adrenergic receptor genotype. *Hypertension*.

[29] Krushkal J, Xiong M, Ferrell R, Sing CF, Turner ST, Boerwinkle E (1998). Linkage and association of adrenergic and dopamine receptor genes in the distal portion of the long arm of chromosome 5 with systolic blood pressure variation. *Human Molecular Genetics*.

[30] Bray MS, Krushkal J, Li L (2000). Positional genomic analysis identifies the *β*
_2_-adrenergic receptor gene as a susceptibility locus for human hypertension. *Circulation*.

[31] Liggett S (1995). Functional properties of human *β*
_2_-adrenergic receptor polymorphisms. *The Neural Information Processing Systems*.

[32] Castellano M, Rossi F, Giacchè M (2003). *β*
_2_-adrenergic receptor gene polymorphism, age, and cardiovascular phenotypes. *Hypertension*.

[33] Herrmann S-M, Nicaud V, Tiret L (2002). Polymorphisms of the *β*
_2_-adrenoceptor (ADRB2) gene and essential hypertension: the ECTIM and PEGASE studies. *Journal of Hypertension*.

[34] Timmermann B, Mo R, Luft FC (1998). *β*-2 adrenoceptor genetic variation is associated with genetic predisposition to essential hypertension: the Bergen Blood Pressure Study. *Kidney International*.

[35] Bengtsson K, Orho-Melander M, Melander O (2001). *β*
_2_-adrenergic receptor gene variation and hypertension in subjects with type 2 diabetes. *Hypertension*.

[36] Bøg-Hansen E, Lindblad U, Bengtsson K, Ranstam J, Melander A, Råstam L (1998). Risk factor clustering in patients with hypertension and non-insulin- dependent diabetes mellitus. The Skaraborg Hypertension Project. *Journal of Internal Medicine*.

[37] Bengtsson K, Orho-Melander M, Lindblad U (1999). Polymorphism in the angiotensin converting enzyme but not in the angiotensinogen gene is associated with hypertension and type 2 diabetes: the Skaraborg Hypertension and Diabetes Project. *Journal of Hypertension*.

[38] (1987). *Treatment of Mild Hypertension*.

[39] (1993). *Treatment of Hypertension in the Elderly*.

[40] Guidelines Sub-Committee (1989). Guidelines for the management of mild hypertension: memorandum from a World Health Organization/International Society of Hypertension meeting. *Journal of Hypertension*.

[41] WHO Study Group (1985). *Diabetes Mellitus*.

[42] Rose GA, Blackburn H, Gillum RF (1982). *Cardiovascular Survey Methods*.

[43] Råstam L, Sjönell G (1991). A new device for measuring blood pressure in adults. *Lancet*.

[44] Gislason T, Almqvist M, Eriksson G, Taube A, Boman G (1988). Prevalence of sleep apnea syndrome among Swedish men—an epidemiological study. *Journal of Clinical Epidemiology*.

[45] Rechtschaffen A, Kales A (1968). *A Manual of Standardized Terminology, Techniques, and Scoring System for Sleep Stages of Human Subjects*.

[46] Vandenplas S, Wiid I, Grobler-Rabie A (1984). Blot hybridisation analysis of genomic DNA. *Journal of Medical Genetics*.

[47] Maqbool A, Hall AS, Ball SG, Balmforth AJ (1999). Common polymorphisms of *β*
_1_-adrenoceptor: identification and rapid screening assay. *Lancet*.

[48] Bao G, Metreveli N, Li R, Taylor A, Fletcher EC (1997). Blood pressure response to chronic episodic hypoxia: role of the sympathetic nervous system. *Journal of Applied Physiology*.

[49] Buxbaum SG, Elston RC, Tishler PV, Redline S (2002). Genetics of the apnea hypopnea index in Caucasians and African Americans: I. Segregation analysis. *Genetic Epidemiology*.

[50] Bartels NK, Börgel J, Wieczorek S (2007). Risk factors and myocardial infarction in patients with obstructive sleep apnea: impact of *β*
_2_-adrenergic receptor polymorphisms. *BMC Medicine*.

